# Cerebellum morphogenesis: the foliation pattern is orchestrated by multi-cellular anchoring centers

**DOI:** 10.1186/1749-8104-2-26

**Published:** 2007-12-03

**Authors:** Anamaria Sudarov, Alexandra L Joyner

**Affiliations:** 1 Department of Cell Biology, New York University School of Medicine, 1st Avenue, NY, NY 10016, USA; 2 Developmental Biology Program, Sloan-Kettering Institute, York Avenue, New York, NY 10021, USA

## Abstract

**Background:**

The cerebellum has a striking morphology consisting of folia separated by fissures of different lengths. Since folia in mammals likely serve as a broad platform on which the anterior-posterior organization of the sensory-motor circuits of the cerebellum are built, it is important to understand how such complex morphology arises.

**Results:**

Using a combination of genetic inducible fate mapping, high-resolution cellular analysis and mutant studies in mouse, we demonstrate that a key event in initiation of foliation is the acquisition of a distinct cytoarchitecture in the regions that will become the base of each fissure. We term these regions 'anchoring centers'. We show that the first manifestation of anchoring centers when the cerebellar outer surface is smooth is an increase in proliferation and inward thickening of the granule cell precursors, which likely causes an associated slight invagination of the Purkinje cell layer. Thereafter, granule cell precursors within anchoring centers become distinctly elongated along the axis of the forming fissure. As the outer cerebellar surface begins to fold inwards, Bergmann glial fibers radiate in towards the base of the immature fissure in a fan shape. Once the anchoring center is formed, outgrowth of folia seems to proceed in a self-sustaining manner driven by granule cell migration along Bergmann glial fibers. Finally, by analyzing a cerebellum foliation mutant (*Engrailed 2*), we demonstrate that changing the timing of anchoring center formation leads to predictable changes in the shape and size of the surrounding folia.

**Conclusion:**

We present a new cellular model of the initial formation of cerebellar fissures with granule cells providing the driving physical force. Both the precise timing of the appearance of anchoring centers at the prospective base of each fissure and the subsequent coordinated action of granule cells and Bergmann glial fibers within the anchoring centers dictates the shape of the folia.

## Background

The cerebellum (Cb) is a morphologically unique brain structure made up of an elaborate set of folia separated by fissures. Recent evidence suggests that the Cb participates in higher order functions, including cognition, emotion and language processing, in addition to its well-documented role in coordinating proprioceptive-motor processing [1,2]. A simple explanation for the evolutionary introduction of folia to the Cb is that it was a means to increase the surface area and thereby accommodate an increase in cell number, which in turn facilitated the acquisition of more complex functional circuits [3]. However, there is increasing evidence that the folia have taken on a role in serving as a platform for organizing Cb circuits. Circuit mapping and physiological studies have demonstrated the specificity of particular sensory-motor tasks to certain folia [4,5]. For example, all spinocerebellar mossy fiber afferents project only to lobules I-V and VIII/anterior IX of the vermis [4,5]. In addition, the embryonic Cb expresses many genes in spatially restricted patterns along the anterior-posterior (AP) axis by the time the afferents enter the Cb, and there is experimental evidence that the later pattern of fissures reflects molecular spatial information within the Cb that climbing fibers respond to [6]. Interestingly, a recent study of Cb foliation in sharks found a better correlation between the degree of foliation and the complexity of displayed behaviors across different species, rather than to their phylogeny [7]. Given that the organization of circuits relates to folia, it is critical to understand how the shape, size and number of folia are regulated during Cb development.

All mammals have a similar basic pattern of ten folia in the medial Cb (vermis), suggesting that foliation may be genetically determined [8-11]. Each folium has three discrete cell layers that surround the white matter and deep nuclei: a thick internal granule layer (IGL) containing granule cells (gcs) and Golgi cells, a monolayer of Purkinje cell (Pc) and Bergmann glial (Bg) cell bodies, and a cell sparse molecular layer containing gc axons (parallel fibers), Pc dendrites, Bg fibers, and basket and stellate cells. We have recently shown that Sonic hedgehog (Shh) secreted by Pcs regulates the number of folia through its influence on gc precursor (gcp) proliferation [12,13]. However, the genes that determine the shape and size of folia are not known.

There have been several proposals for how mechanical forces could induce fissure formation [9,14-16]. One intriguing suggestion is that a subset of Pcs anchors the cortex to the underlying white matter via the Pcs' axons at positions that define the base of fissures [9]. Alternatively, differential rates of gcp proliferation, with highest rates at the base of the fissures, have been suggested to underlie the postnatal growth phase of folia [14]. However, evidence for distinct Pc morphologies or differential gcp proliferation at the embryonic stage when fissures form has not been reported.

As a first step in identifying the key cellular events that partition the Cb into distinct folia, we have identified a reproducible series of cellular changes that the three major Cb cell types undergo during initial formation of fissures. We also demonstrate that the timing of these cellular changes governs folial shape by analyzing a mouse Cb foliation mutant. We propose a model for Cb foliation whereby changes in gcp behavior drive formation of 'anchoring centers' at the base of each fissure consisting of Pcs, gcs and Bg, and then folia outgrowth continues by a self-sustaining process involving the coordinated action of gcs and Bg.

## Results

### The base of each fissure is fixed while the lobes grow outward

**Figure 1:**
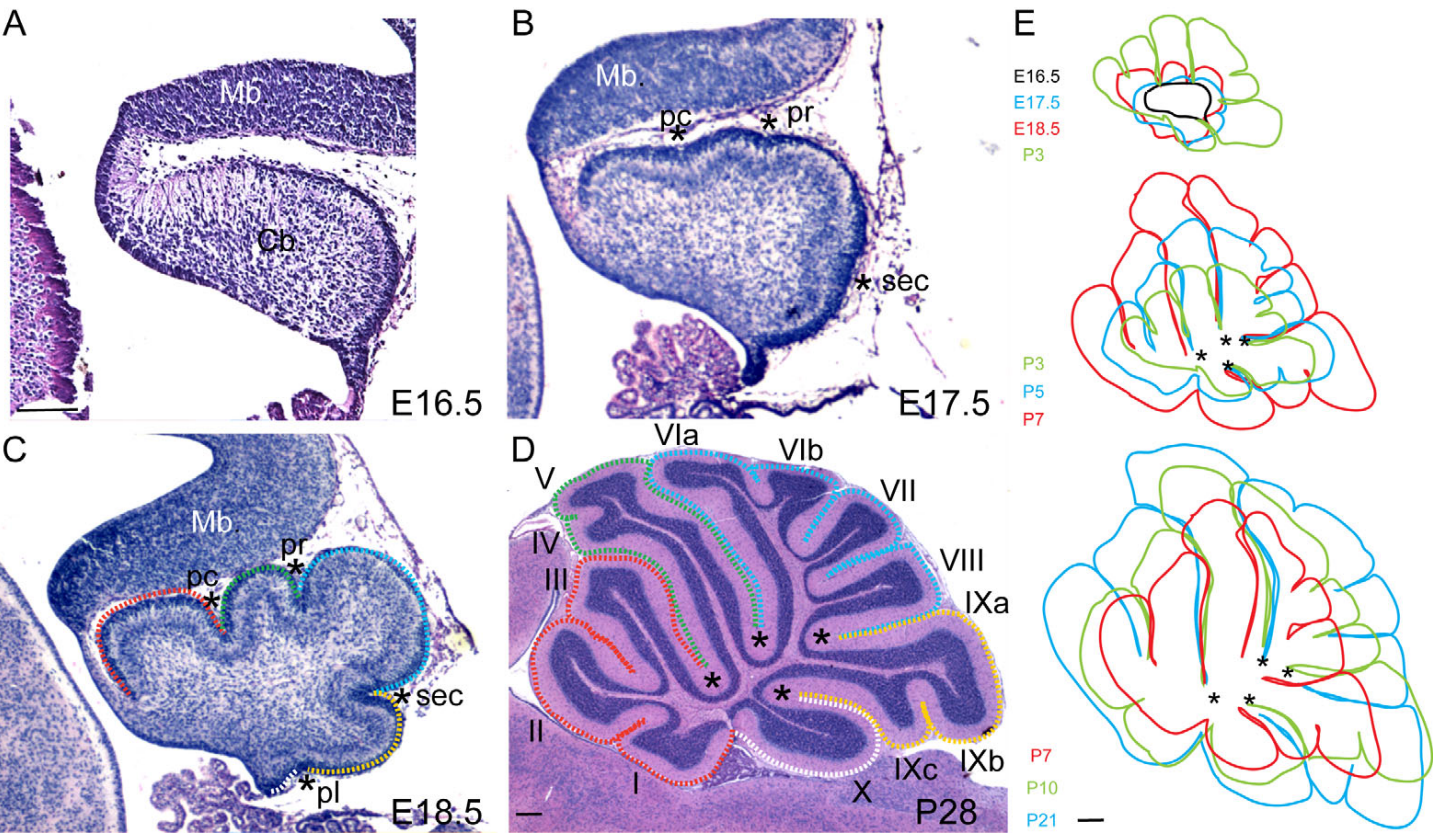
Cerebellar foliation is executed by anchoring the base of the fissures and by folia lengthening. **(a, b) **The Cb vermis primordium at E16.5 is a smooth structure (a), which, at E17.5 (b), is divided into four lobes by three fissures (asterisks). **(c) **At E18.5 the four principal fissures (asterisks) and five cardinal lobes (dotted outlines) are evident. The cardinal lobes are designated as anterobasal (red outline), anterodorsal (green outline), central (blue outline), posterior (yellow outline) and inferior (white outline). **(d) **The adult Cb has ten lobules that develop from the cardinal lobes, marked by colored outlines in (c, d) (I-X, principal fissures are marked by asterisks). **(e) **Superimposition of midline vermis tracings from E16.5 to P21 show that the folia grow by lengthening, while the bases of the fissures are largely fixed in position. Mb, midbrain; Cb, cerebellum; pc, preculminate; pr, primary; sec, secondary; pl, posterolateral fissure. Scale bars: (a-c) 300 μm; (d) 500 μm; (e) 600 μm.

If initiation of foliation is dependent on Pc anchor points, then the base of each fissure should be fixed relative to the core of the Cb and folia should bulge out in between the anchor points. To address whether this is the case, we superimposed tracings of the outline of midsagittal sections of the mouse vermis at stages between embryonic day (E)16.5 and postnatal day (P)21 (see Materials and methods). In outbred Swiss Webster (SW) mouse embryos, the outer surface of the mouse cerebellar vermis primordium was found to be smooth at E16.5 (Figure 1a). By E17.5, however, the surface was already divided by three slight indentations representing three of the four principal fissures: preculminate, primary, and secondary (Figure 1b, asterisks). At E18.5, the fourth principal fissure (posterolateral) also was evident and the other fissures were deeper (Figure 1c, asterisks). The five cardinal lobes seen in all mammals, and that form between the principal fissures, were thus apparent at E18.5. From anterior to posterior, they are called the anterobasal, anterodorsal, central, posterior and inferior lobes (Figure 1) [9].

Additional fissures (non-principal) successively divided the cardinal lobes into lobules until P7 in SW mice. The anterobasal lobe was the first to be divided by a non-principal fissure at P0 in SW mice, giving rise to lobules I/II and III [8,17]. In some strains of mice an additional shallow fissure subsequently demarcates lobule I as separate from lobule II, but this was rarely seen in SW mice. In most mouse strains, an additional partial fissure forms in the anterodorsal lobe to partially divide lobules IV and V by a shallow fissure, and this was observed by P5 in the majority of SW mice examined (data not shown). The first subdivision of the central lobe was seen at P1 when the prepyramidal fissure demarcates lobules VII and VIII (data not shown). By P3 the central lobe was further subdivided by the posterior superior fissure into lobules VI and VII (data not shown). In most strains lobule VI is further subdivided by a fissure to form sublobules VIa and VIb, which was seen at P5 in SW mice (data not shown). The posterior lobe is subdivided in some mouse strains to form sublobules IXa, IXb, and IXc. In SW mice the fissure producing IXb and IXc was first apparent at P3, whereas the fissure between IXa and IXb was never seen (data not shown). The inferior lobe is not further divided in mice and is referred to as lobule X (Figure 1d).

By superimposing images of the Cb surface at successive times during the foliation process, we found that indeed the base of each fissure remained in a relatively fixed position and the folia grew outward (Figure 1e). There was a slight outward shift in the positions of the bases of the fissures after P7, likely as a result of the expansion of the white matter and cortex, especially in the central and posterior lobe. Our results are consistent with the base of the fissures functioning as anchors for Cb foliation.

### Folding of the Purkinje cell layer predicts the positions of fissures

**Figure 2:**
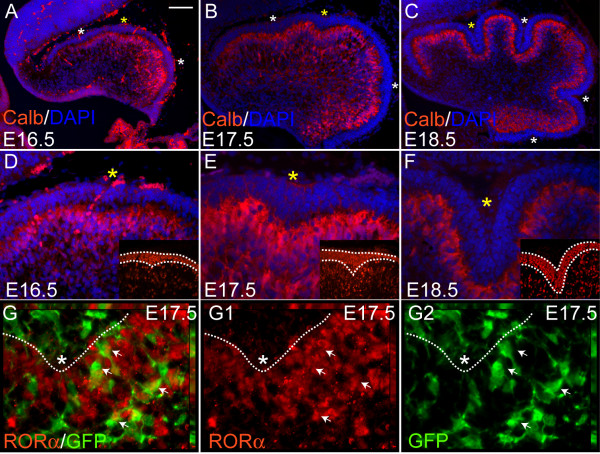
Purkinje cell layer folding indicates the future positions of the base of each principal fissure. (**a, d**) At E16.5 the mouse Cb has a smooth surface, but anti-Calbindin immunostaining (red) shows a multilayer of Pcs with invaginations in the areas where fissures will form (asterisks). Yellow asterisk indicates the fissures shown in (d, e, f). (**b, c, e, f**) At E17.5 (b, e) and E18.5 (c, f) both the Pc layer and outer surface invaginate (foliate) simultaneously. Anti-Pax6 immunostaining shows accumulation of the gcps in the EGL, above the Pc layer invagination at E16.5 and E17.5 (inset in (d, e)), whereas by E18.5 the EGL is similar in thickness at the base and at the crown of the folia (inset in (f)). (**g-g2**) In *R26-CreER; R26R-eYFP *animals, *Cre *is expressed ubiquitously in precursors. Upon administration of tamoxifen at E12.5, *Cre*ER translocates to the nucleus, where it recombines the floxed STOP signal in the *R26-eYFP *locus. Sagittal sections of E17.5 Cb show that some fate mapped cells (green) colocalize with anti-RORα (red) and are, therefore, Pcs. Marked Pcs (white arrows) have round cell bodies and have extended their axons in various directions. Scale bars: (a-c) 100 μm; (d-f) 40 μm; (g-g2) 15 μm.

Since it has been suggested that Pcs, the largest cells in the Cb and sole output of the cortex, are responsible for anchoring the base of the principal fissures, we reasoned that changes in the organization and/or morphology of Pcs might precede formation of fissures [9]. To visualize Pc morphology during initiation of foliation, we stained sagittal Cb sections for the Pc marker Calbindin before (E16.5) and during the initiation of foliation (E17.5 and E18.5) [18]. Anti-Calbindin immunostaining detected a diffuse multilayer of similarly shaped Pcs along the AP axis at E16.5 (Figure 2a). Strikingly, at E16.5 the Pc multilayer was slightly folded inwards (Figure 2a, d, asterisks) at the sites where the three principal fissures formed at E17.5 (Figure 2b, e). By E18.5 all four principal fissures were visible (Figure 2c, f). Interestingly, the invaginations of the Pc multilayer were complementary to sites of inward accumulation of gcps in the external granular layer (EGL) detected by anti-Pax6 immunostaining (insets in Figure 2d–f).

Since Pcs around the base of the fissures at P18 have been found to orient their dendrites towards the base of the fissure, we were curious whether the morphology of individual Pcs was distinct where the fissures emerge at E16.5-E18.5 [19]. We utilized the CreER/loxP based genetic inducible fate mapping (GIFM) approach to mark cells and visualize the cell body and its processes [20]. We used a *R26-CreER *line (Y Cheng and AL Joyner, in  preparation) in combination with a reporter allele that expresses enhanced yellow fluorescent protein (eYFP; *R26-eYFP*), and induced Cre activity in all cerebellar cell types by administering tamoxifen at E12.5 (Figure 2) [21]. At E16.5 and E17.5, marked cells (YFP positive) were detected in the EGL, as well as in deeper layers of the Cb (Figure 2g–g2, and data not shown). Double immunostaining of cells for anti-green fluorescent protein (GFP) and the Pc nuclear marker anti-RORα identified fate mapped Pcs (Figure 2g–g2) [22]. Within the deeper layer of the Cb cortex, marked cells positive for RORα had small round cell bodies and processes that were extended in various directions (Figure 2g–g2, white arrows). Consistent with previous reports, immature Pcs had numerous randomly oriented projections, and furthermore, Pcs in the emerging fissures had the same morphology with randomly oriented processes (Figure 2g–g2) [9,23,24]. In conclusion, the folding of the Pc multilayer with associated accumulation of gcps in the EGL, rather than a change in individual Pc morphology, precedes fissure formation at the outer surface of the Cb.

### Purkinje cell maturation is synchronized with fissure lengthening

**Figure 3:**
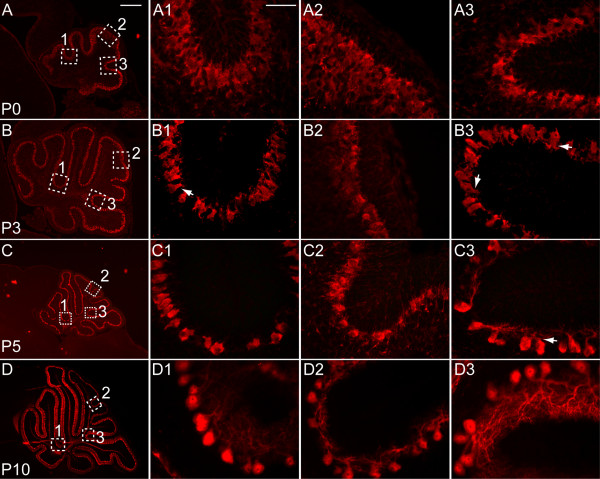
Maturation of Pcs is concomitant with fissure formation. **(a) **At P0, anti-Calbindin immunostaining (red) shows that Pcs are organized in a multilayer throughout the Cb. In the principal (a1, a3) and non-principal (a2) fissures, the Pcs appear equally undeveloped, with small bodies and no apparent dendrites. **(b) **By P3, Pcs in the principal fissures (b1, b3) are arranged in a monolayer with distinct cell bodies and an apical dendrite (b1, arrow), whereas, in the non-principal fissure (b2), Pcs remain in a multilayer. The secondary fissure contains the most mature Pcs in the Cb, with dendritic branches beginning to form on the apical stem dendrite (b3, arrow). **(c) **At P5, Calbindin staining is more intense and more uniform. In all principal fissures, the Pc bodies have increased in size, and dendritic branches have begun to form (c1, arrow). In the secondary fissure (c3), Pcs are more mature than in other principal fissures, exhibiting more and longer dendritic branches (c3, arrow). Pcs in the non-principal fissure become organized into a monolayer (c2). **(d) **At P10, in all fissures, the number of secondary and tertiary dendritic branches in the Pcs continues to increase and the branches themselves continue to increase in length: (d1) preculminate fissure; (d2) prepyramidal fissure; (d3) secondary fissure. Scale bars: (a, b) 300 μm; (c, d) 600 μm; (a1-a3, b1-b3, c1-c3, d1-d3) 100 μm.

Since a majority of the maturation of Cb cells occurs during the period of foliation, this raises the question of whether the two processes are linked. In the rat Cb, Pcs located in the anterior (I-V) and posterior (IX-X) folia have been found to mature earlier than Pcs in the central folia [25]. Consistent with the timing being similar in mouse, Pcs in the anterior and posterior lobules of the mouse vermis also are more mature at P7 [26]. To determine whether Pc development proceeds in synchrony with fissure outgrowth, we constructed a developmental profile of the Pcs in the principal fissures compared to later forming non-principal fissures from P0 to P21, using anti-Calbindin immunostaining to visualize Pcs (Figure 3, and data not shown). We judged Pc maturation based on an increase in cell body size and development of dendrites. Initially, the single apical stem dendrite of each Pc appears as an apical swelling, and then the dendrite extends outward and forms a large number of mature dendritic branches [9]. At P0, with the exception of the Pcs in the central lobe, Pcs in the vermis had small cell bodies with an apical swelling and no obvious dendrites, and were organized in a two to three cell thick layer. In contrast, Pcs in the central lobe lacked an apical swelling and were organized into a four to six cell thick layer (Figure 3a–a3). There was no obvious difference in maturation of Pcs at the base versus elsewhere in the forming principal fissures. By P3, Pcs throughout the principal fissures were arranged in a monolayer (Figure 3b1, b3, and data not shown), and the cell bodies had grown in size and a single apical stem dendrite had formed (Figure 3, arrows in 3b1, b3). Curiously, Pcs at the base and the sides of the secondary fissure, which is one of the four principal fissures (between lobules VIII and IX) appeared more mature than elsewhere as they had the largest cell bodies and had begun to form primary dendritic branches (Figure 3b3, arrows).

In contrast to Pcs in the principal fissures at P3, the Pcs in the posterior-superior non-principal fissure, which begins to form at P2, remained in a multilayer, with small cell bodies and no apical dendrite (Figure 3b2). At P5, anti-Calbindin immunostaining was more uniform and stronger in all fissures, perhaps reflecting a progression in Pc differentiation (Figure 3c–c3). In all principal fissures at this stage (Figure 3c1, c3), the Pc bodies had increased in size and primary dendritic branches were evident. Similar to P3, the most mature Pcs were found in the secondary fissure as they had elaborate secondary and tertiary dendritic branches (Figure 3c3, arrow). In the less mature posterior superior fissure at P5, a Pc monolayer had formed (Figure 3c2). At P10, Pcs in the posterior superior fissure had extended and increased their number of secondary and tertiary dendritic branches (Figure 3d2). Pcs in all fissures continued to elaborate the number of their secondary and tertiary dendritic branches after P10 (Figure 3d–d3, and data not shown). In summary, development of Pcs throughout a given fissure proceeds in synchrony with the maturation of the fissure, although Pcs within the secondary fissure are more developmentally advanced from P0 until about P10 than in other principal fissures, suggesting there is no causative link between Pc maturation and formation of fissures.

### Granule cell precursors in emerging fissures have a shorter mitotic index than other granule cell precursors and accumulate as inward invaginations

**Figure 4:**
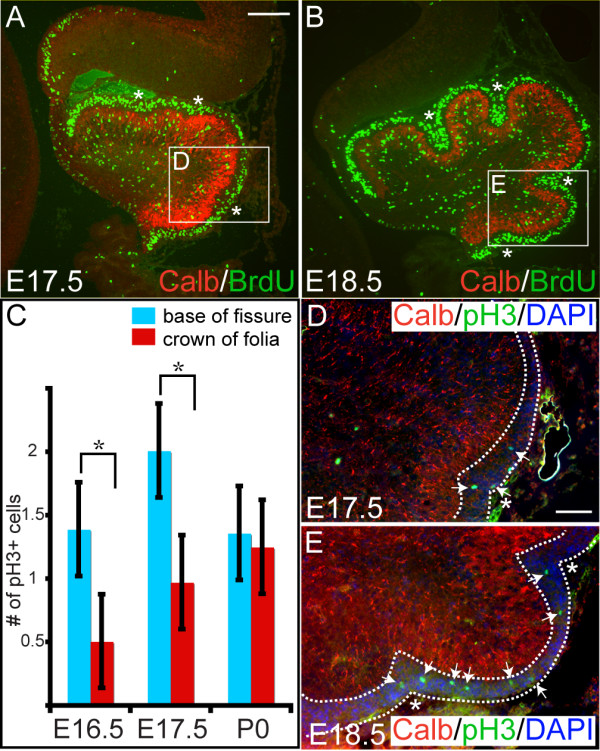
Gcps in the emerging fissures have a shorter mitotic index than other gcps. **(a, b) **Medial sagittal sections of E17.5 (a) and E18.5 (b) embryos treated for approximately 20 minutes with BrdU. Anti-BrdU (green) immunostaining shows uniform BrdU incorporation throughout the EGL (asterisks depict locations where fissures will form). **(c) **Quantification of the number of pH3 positive gcps in the region where the EGL is thicker (blue bars) versus the intervening thinner regions (red bars). Bar height indicates the mean value of each data set, and error bars indicate standard error. An asterisk indicates statistically significant differences between the two regions (*p *< 0.001 for E16.5, *p *< 0.0001 for E17.5). **(d) **Anti-pH3 immunostaining at E16.5 (data not shown) and E17.5 indicates that there are more gcps in mitosis in the regions where future fissures will form (areas between dotted outline) than in other regions. **(e) **By E18.5 pH3-positive cells are distributed equally throughout the EGL (between dotted lines). Anti-Calbindin was used to mark the Pc layer. Arrows indicate pH3 positive cells in the EGL. Scale bars: (a, b) 100 μm; (d, e) 50 μm.

Unlike the Pcs and Bg that are born in the ventricular layer and then migrate radially to form the Pc layer, when gcps leave the ventricular layer (rhombic lip) between E12.5 and E15.5 in mice they migrate over the surface of the Cb to form the EGL and continue proliferating until about P16 [27]. Once gcs become postmitotic, they migrate through the molecular layer and past the Pc layer to form the IGL. A distinct feature of gcps in the EGL that we detected as early as E16.5, one day prior to the first folding of the outer surface of the Cb, was a V-shaped thickening of the EGL, specifically in the positions where the first three principal fissures will emerge (Figure 2a, d). One possible mechanism for the increased number of gcps at these positions is an increase in the rate of proliferation. We therefore examined gcp proliferation at the onset of Cb foliation using markers for different stages of the cell cycle. To mark gcps in the DNA replication phase (S phase) at E17.5 and E18.5, bromodeoxyuridine (BrdU) was administered approximately 20 minutes prior to analysis of the embryonic Cb (Figure 4a, b). This resulted in marking of approximately 26% of cells within the EGL and did not reveal an obvious difference in the number of cells incorporating BrdU at the bottom or the crown of the folia at E17.5 or E18.5 (Figure 4a, b).

In order to mark a shorter phase of the cell cycle than S phase, nuclei undergoing the G2/mitosis phase were marked using anti- phosphohistone 3 immunostaining. In contrast to the seemingly uniform BrdU labeling throughout the AP axis of the EGL, quantification of pH3 positive gcps (Figure 4c) revealed that pH3 positive gcps were more frequently found at the sites where the first three principal fissures were emerging then elsewhere at E16.5 and E17.5 (Figure 4c, d, arrows; data not shown). However, this was not the case for these fissures at E18.5 when pH3 positive gcps did not reveal a significant difference in the distribution throughout the EGL (Figure 4c, e, arrows; data not shown). This result suggests that gcps in the area of the emerging fissures transiently divide more often than other gcps during initiation of fissure formation due to a shorter cell cycle. Furthermore, possibly because there is less resistance in the Cb cortex than in the overlying basal lamina, the gcps invaginate inwards to produce the first morphological manifestation of the base of the fissures. By E18.5, the width of EGL at the base was only slightly thicker than at the crown of principal fissures, consistent with our observation that cells in the base of the fissures no longer had an obviously higher mitotic index (Figures 2f and 4b). Moreover, during establishment of the non-principal fissures at later stages, gcps were found to accumulate where the fissures later formed, indicating that this process is conserved for all cerebellar fissures (data not shown).

### Granule cell precursor morphology and organization is distinct at the base of emerging fissures

**Figure 5:**
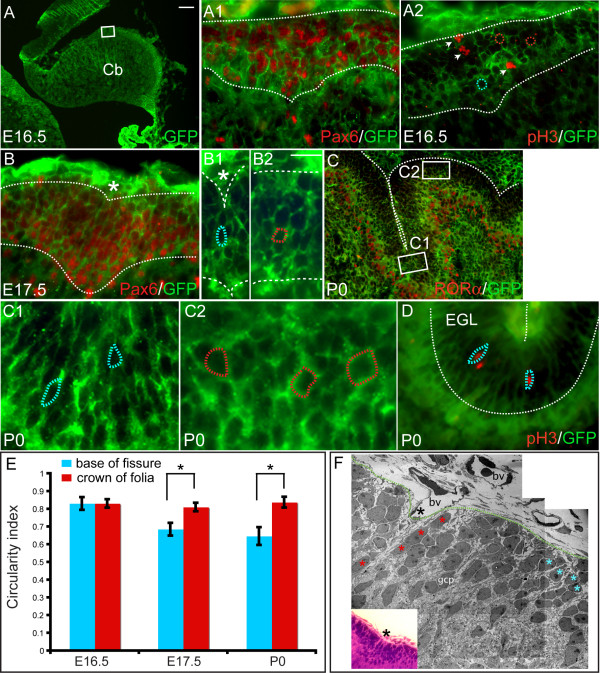
Anchoring center formation involves changes in gcp shape. Sagittal sections of the Cb of *CAG::GPI-eGFP *transgenic mice to label cell membranes. **(a-a2) **At E16.5, anti-GFP immunostaining reveals round gcps throughout the AP axis of the EGL. (a1) Anti-Pax6 (red) and anti-GFP (green) immunostaining confirms that cells in the outermost layer are gcps. (a2) Anti-pH3 and anti-GFP immunostaining shows that round gcps are proliferating (arrowheads). White box in a indicates the region in a1, a2. **(b-b2) **At E17.5, anti-GFP and anti-Pax6 immunostaining reveals elongated gcps (blue dotted line) at emerging anchoring centers, in contrast to the more round gcps (red dotted line) in the adjacent areas (b2). Asterisk indicates the fissure. **(c-c2) **The gcp shape differences are more evident in the fissures at P0. Elongated gcps (blue dotted line) are found at the base of the fissures and appear organized; their longitudinal axes aligned with the plane of the fissure. At the crown of the folia, gcps are round and less organized (red dotted line). **(d) **Anti-pH3 immunostaining indicates that the elongated gcps at the base of the fissure are proliferating at P0. **(e) **Circularity index of the gcps (blue at the base of fissure, red at the crown of the folia). Bar height indicates the mean value of each data set, and error bars indicate standard error. An asterisk indicates statistically significant differences in ci between the base of the fissure and the crown of the folia for each data set (*p *< 0.0001). **(f) **Electron micrograph of the emerging primary anchoring center at E17.5 confirms that gcps located in the fissure (red asterisks) are elongated and organized in a plane parallel to the fissure (black asterisk). Gcps located between the fissures are more rounded (blue asterisks). Inset is a bright field image of the same area. White dotted lines indicate EGL. Bv, blood vessel; gcp, granule cell precursors. Scale bars: (a) 100 μm; (c) 50 μm; (a1, a2, b, d) 15 μm; (b1, b2, c1, c2) 10 μm.

Cell shape changes have been implicated as a driving force in formation of grooves or invaginations in many model systems [28]. Since there are no obvious differences in the shape or orientation of Pcs at the base of the fissure between E16.5 to E18.5 (Figure 2), we asked whether changes in gcp morphology accompany the initial formation of fissures (Figure 5). We characterized the shape of the gcp cell bodies by examining GFP expression in transgenic mice that ubiquitously express a cell membrane localized GFP (*CAG::GPI-eGFP*) [29]. To quantify differences in cell morphology, we determined the circularity index (ci; Figure 5e) of GFP+ gcps in the first three emerging fissures (within the invagination) at E16.5 (Figure 5a, a1), E17.5 (Figure 5b–b2) and P0 (Figure 5c, d) compared to gcps between the fissures (at the crown) [30]. The EGL was identified as the outer most five cell layers at the crowns of the folia and seven cell layers at the base of the fissures by double immunostaining for anti-GFP and anti-Pax6, a gcp marker or anti-RORα, a Pc marker (Figure 5a1, b, c). At E16.5 when the outer surface of the Cb is smooth, proliferating gcps throughout the AP axis of the midline vermis had a round shape (Figure 5a2, e; ci = 0.830 at the crown of folia, ci = 0.8 at the future base of fissures) [31]. At E17.5 when the outer surface begins to invaginate, gcps in these emerging fissures were significantly less round (Figure 5b1, e; ci = 0.685) than gcps at the midpoint between fissures (Figure 5b2, e; ci = 0.810, *p *< 0.0001). Furthermore, at E17.5 the axis of elongation of gcs at the base of the fissures was uniformly parallel to the fissure. In contrast, there was no preferential orientation of the longitudinal axes of gcps at the crown of folia (Figure 5b2, and data not shown). At P0, the difference in gcp shape between the bases of the three principal fissures (Figure 5c, e; ci = 0.646) and the crowns (Figure 5d, e; ci = 0.837) was even more evident (*p *< 0.0001). Furthermore, we found that at E17.5 and P0 elongated gcps are mitotic since they were positive for anti-pH3 marker (Figure 5d, and data not shown).

To further characterize gcps in emerging fissures and to confirm our findings in *CAG::GPI-eGFP *transgenic mice, we used electron microscopy to analyze sagittal sections at E17.5 (Figure 5f). The gcps located at the base of the fissure were clearly elongated (ci = 0.65) and their longitudinal axes were parallel to the fissure (Figure 5f). In contrast, gcps located five to seven cells away from the base of the fissure were more round (ci = 0.78) without any consistent alignment of their axes (Figure 5f). Interestingly, gcps found in between these two positions had an intermediate phenotype in terms of cell shape (ci = 0.76), but they lacked an organized orientation of their longitudinal axes. Thus, gcp cell body elongation and an inward accumulation of gcps due to an increased proliferation are clear hallmarks of the emergence of the fissures and these cellular changes may drive the inward folding of the Pc layer. We therefore define the entire region undergoing these unique morphogenetic changes as an 'anchoring center' for each fissure.

### The onset of granule cell differentiation is concomitant with cerebellar foliation

We next explored whether the migration and differentiation of gcps from the EGL to the IGL is different in fissures from the rest of the folia. Gcps proliferate only in the outer EGL (oEGL) and then move to the inner EGL (iEGL) when they begin to differentiate. Postmitotic gcs within the iEGL extend parallel fibers along the medial/lateral axis and undergo nuclear translocation along one parallel fiber before extending a radial process and descending along Bg fibers past the Pcs to form the IGL [9,32,33]. The gc parallel fibers constitute part of the molecular layer. Anti-p27/Kip1 and anti-NeuN were used to mark differentiating gcs both in the iEGL and during their migration to the IGL (Additional file 1). The earliest a diffuse IGL layer could be detected using these markers was at P1 (Additional file 1a, a1). As has been reported, we found the IGL to be thinner at the base of the fissures than the sides and thickest at the crown of the folia at all stages analyzed between P3 and P21, but not at P1 (Additional file 1b–b2, c–c2, and data not shown) [9].

**Figure 6:**
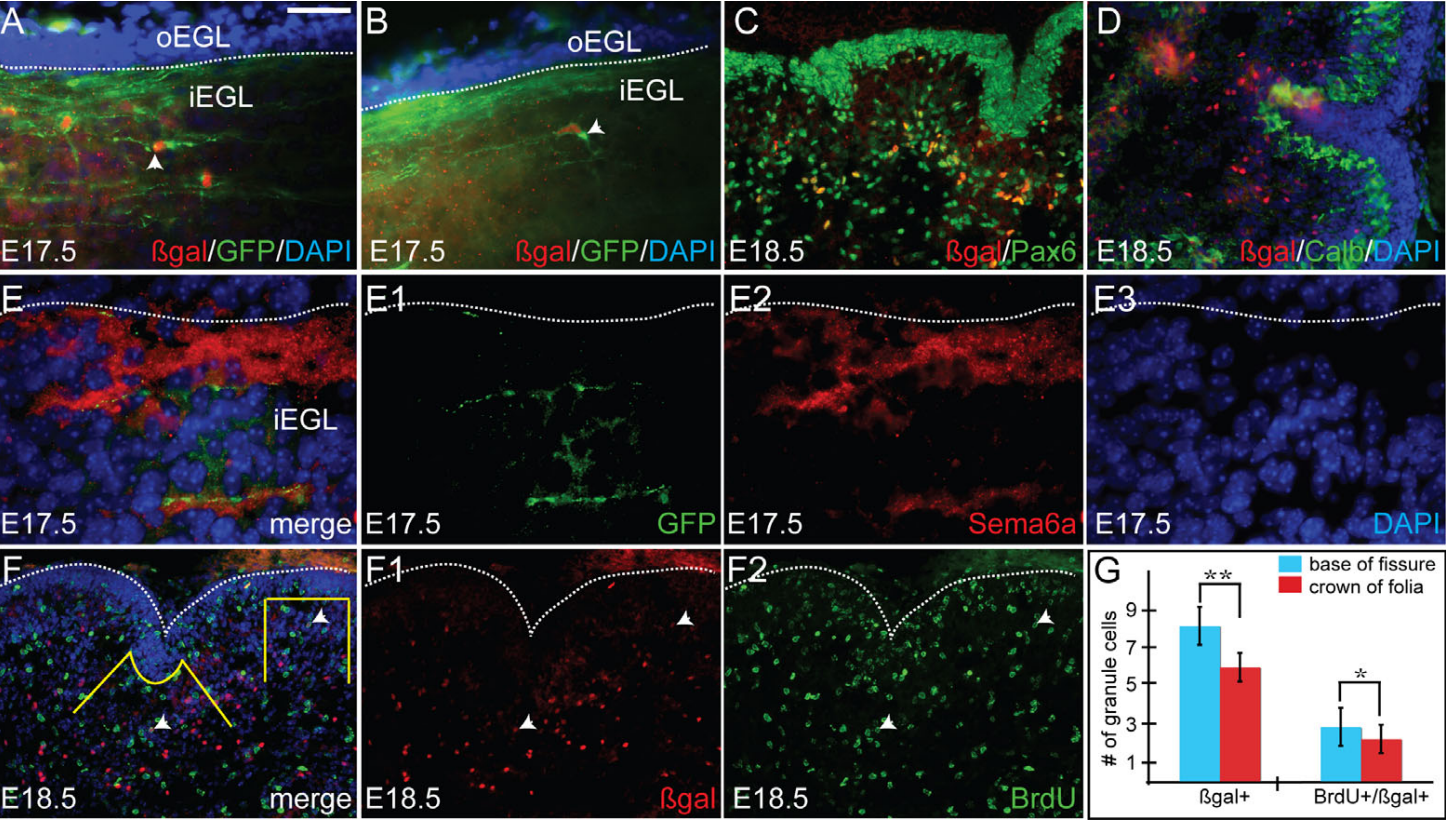
Granule cell differentiation occurs simultaneously with the onset of cerebellar foliation. *Math1-CreER; Tau-STOP-myrGFP-IRES-nLacZ *animals were administered tamoxifen at E15.5. Permanent *myrGFP *and nuclear *LacZ *expression are initiated once cells become postmitotic. Gcps (DAPI positive) proliferate in the oEGL. **(a, b) **Coronal sections of E17.5 Cb show that fate mapped gcs (red) are located in the inner layer of the EGL (iEGL). (a) Marked gcs (arrowhead) have round cell bodies and have extended their axons (parallel fibers; green). (b) In the layers below the iEGL, a 90° elongated cell body rotation (arrow) of some granule cells can be visualized, in addition to a developing descending radial fiber. **(c) **Anti-Pax6 (green) and anti-βgal (red) double immunostaining confirms that the fate mapped cells are granule cells. **(d) **Anti-Calbindin (green) and anti-βgal (red) double immunostaining reveals that fate mapped cells are found below the Pc layer. **(e-e3) **At E17.5, fate mapped cells are positive for Sema6a, a marker for translobular migrating granule cells. Dotted line indicates the border between oEGL and iEGL. **(f-f2) **Double immunostaining for anti-BrdU and anti-βgal reveals that gcs differentiate at a slightly higher rate at the base of the fissure than at the crown of the folia. Yellow lines in (f) depict the area from which BrdU/βgal positive cells were counted. Arrows indicate BrdU/βgal positive gcs. **(g) **Bar graphs depict quantification of BrdU/βgal positive gcs at the base of fissure (blue bars) versus the crown of the folia (red bars). Bar height indicates the mean value of each data set, and error bars indicate standard error. An asterisk indicates statistically significant differences between the base of the fissure and the crown of the folia for each data set (*p *< 0.006 for βgal; *p *< 0.05 for BrdU/βgal). Scale bars: (a, b) 50 μm; (c, d, f) 100 μm; (e) 25 μm.

Since the lack of gc differentiation before P1 (that is, during emergence of the principal fissures) could be due to lack of expression of p27/NeuN rather than an absence of differentiation of gcs, we utilized GIFM to mark postmitotic gcs independent of the timing of expression of gc marker genes. Given that all gcps express the transcription factor Math1, we used a *Math1-CreER *transgenic line in combination with a postmitotic neuron reporter allele (*Tau-lox-STOP-myrGFP-nlacZ*), and induced Cre activity in embryonic gcp by administering tamoxifen at E15.5 (Figure 6) [34,35]. The *Tau *reporter allele initiates permanent expression of membrane localized GFP and nuclear lacZ (nlacZ) in neurons as they begin to differentiate. Coronal sections at E17.5 revealed marked gcs (GFP/nlacZ double positive) both in the iEGL, based on their position below the outer three to five cell layers of the EGL and labeling with Semaphorin6a (Sema6a), a marker for gcs undergoing nuclear tangential migration (Figure 6a, b, e–e3), as well as in deeper positions (Figure 6a, b, e-e3) [36,37]. Double immunohistochemistry for anti-Pax6 and anti-GFP on sagittal sections at E18.5 confirmed that all the marked cells were indeed gcs (Figure 6c). The combination of nlacZ and myrGFP immunostaining revealed that the gcs within the iEGL had a round cell body and parallel fibers (Figure 6a, arrow). Within the deeper layer of the Cb, below the iEGL, some gcs had elongated cell bodies parallel to the surface of the Cb and a descending radial fiber (Figure 6b, arrow). Furthermore, we found no obvious differences in morphology and orientation of the gc bodies, or their parallel fibers at different AP positions, suggesting that the process of gc differentiation is similar at the base of fissures and crowns of folia. By E18.5 descendents of the gcps marked at E16.5 could be seen accumulating in a broad and diffuse layer below the Pcs (anti-Calbindin positive cells; Figure 6d), consistent with our detection of a sparse EGL at P1 using anti-p27/Kip1 or NeuN (Additional file 1a, and data not shown).

To address whether gcps differentiation occurs at the same time at the base of the fissures versus the crown of the folia, we used GIFM to mark gcps with tamoxifen at E15.5 followed by a BrdU pulse at E16.5, and analysis of midsagittal Cb sections at E18.5 (Figure 6f–f2). Quantification of cells labeled for anti-βgalactosidase (anti-βgal) in the base of the first three fissures to form versus the crown of the adjacent folia revealed that gcs differentiate at both positions at E16.5 (Figure 6g). Interestingly, we found more differentiated gcs at the base of the fissures (mean = 8.18 cells below a 10 micron region of the iEGL; Figure 6g) than at the crowns of the folia (mean = 6 cells below a 10 micron region of the iEGL; *p *< 0.006; Figure 6g). Quantification of anti-βgal and anti-BrdU double positive gcs showed a tendency toward an increase in the number of differentiated gcs born at E16.5 at the base of the fissures (mean = 2.85 cells below a 10 micron region of the iEGL; Figure 6g) versus the crown of the folia (mean = 2.22 cells below a 10 micron region of the iEGL; *p *< 0.05; Figure 6g). Moreover, we found double positive (BrdU/βgal) gcs only in the most medial sections of the vermis (within 80–100 μm of the midline), where the three principal fissures are the longest at E18.5. In conclusion, gcs begin their differentiation program as early as E17.5 in the most medial Cb, coinciding with the position where foliation is first observed, and preferentially at the base of the emerging fissures.

### Bergmann glial fibers fan out from a single central point at the base of fissures

**Figure 7:**
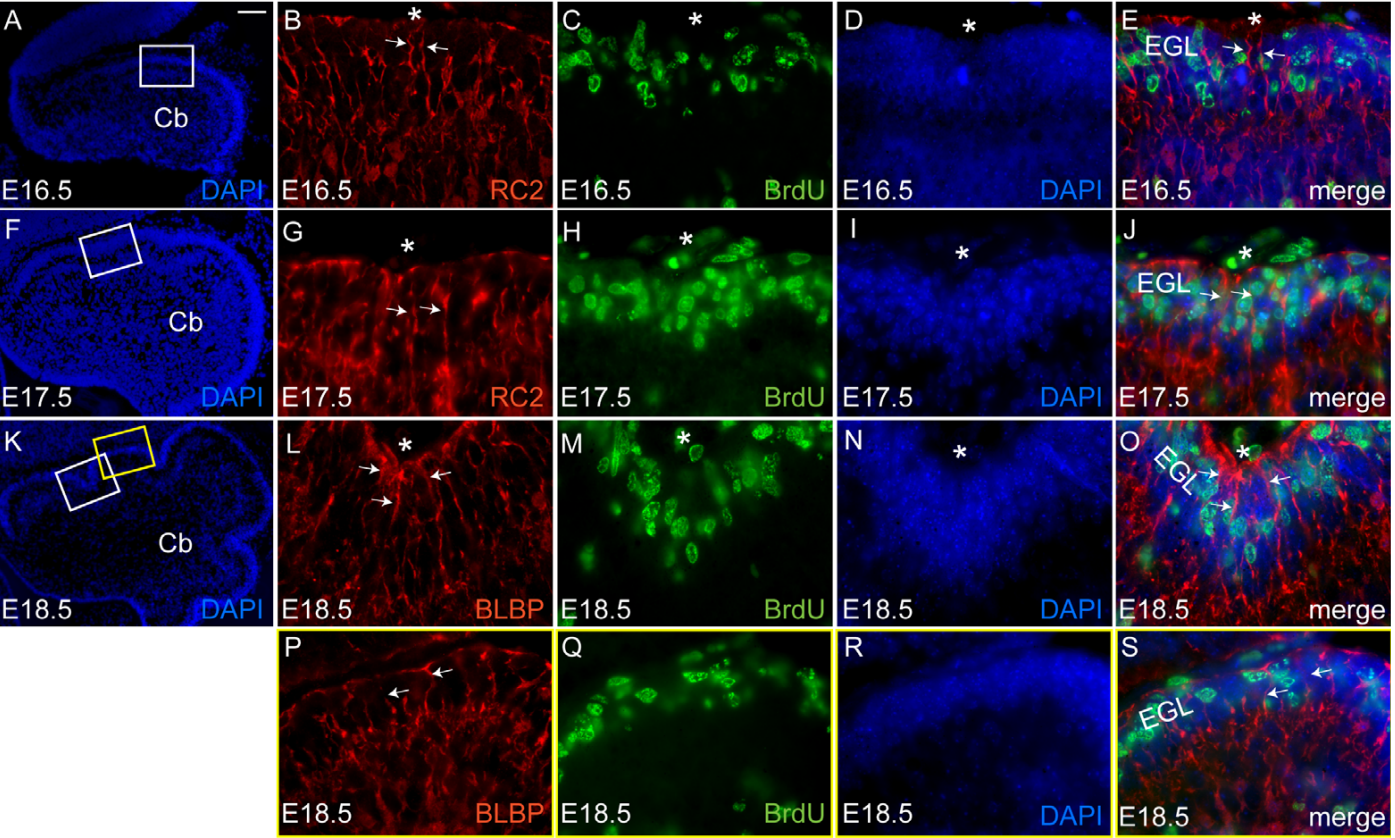
Bg fibers converge on the anchoring centers. **(a-e) **Anti-RC2 (red) and anti-BrdU (green) immunostaining of midsagittal section of the E16.5 Cb. (b) Bg fibers (arrows) located around the anchoring centers (asterisk) are oriented perpendicular to the outer surface of the Cb. **(f-j) **Anti-RC2 (red) and anti-BrdU (green) immunostaining of a midsagittal section of the E17.5 Cb reveals that, at the onset of fissure formation, Bg fibers (arrows) remain parallel to each other. **(k-s) **Anti-BLBP (red) and anti-BrdU (green) immunostaining of midsagittal sections of the E18.5 Cb, at the base of fissure (l-o) and at the crown of the folium (p-s), reveal a unique orientation of Bg fibers (arrows). (l) Bg fibers (arrows) in the anchoring centers fan out from a single point (asterisk). (p) Conversely, the Bg fibers (arrows) at the crown of the folia remain aligned perpendicular to the pial surface of the Cb. Cb, cerebellum; EGL, external granular layer. Scale bars: (a, f, k) 100 μm; (b-e, g-j, p-s) 15 μm.

Each Bg cell projects a single fiber to the pial surface that forms a specialized structure called a glial endfoot that together with the basal lamina maintains the integrity of the Cb [9,38,39]. Furthermore, Bg provide a scaffold for Pc dendritic outgrowth and for gc migration [9,40-43]. Thus, Bg fibers could contribute to the emergence or maturation of fissures. To investigate whether Bg play a such role in anchoring centers, we used anti-RC2, anti-brain lipid binding protein (anti-BLBP) and anti-glial fibrillary acidic protein (anti-GFAP) immunostaining to visualize the orientation of the Bg fibers from E16.5 to P14 (Figure 7, and data not shown) [44-47].

In order to establish the orientation of the Bg fibers in relation to the first manifestations of anchoring centers (accumulation of gcps and invagination of the Pc layer), we double labeled for Bg and gcps or Pcs. To label gcps, we utilized a 20 minute BrdU pulse to identify the proliferative layer of the EGL (anti-Pax6 positive in control experiments; see Additional file 2). At E16.5 and E17.5 anti-RC2 immunostaining revealed that Bg fibers were oriented parallel to each other and perpendicular to the Cb outer surface, even in the emerging fissures where gcps accumulated and the Pc layer invaginated (Figure 7a–j). Interestingly, at E18.5 (Figure 7k–s), anti-BLBP immunostaining revealed that the Bg surrounding the emerging principal fissures projected their fibers to a single point at the base of the fissure (Figure 7l, asterisk). In contrast, the remaining Bg fibers were oriented nearly parallel to each other and aligned perpendicular to the pial surface of the Cb (Figure 7p). At later stages, this specific organization of Bg fibers at the base and the sides of the fissure was more pronounced (data not shown). Our analysis suggests that the glial endfeet of Bg fibers surrounding the base of the emerging fissures form a hub from which the fibers fan out. This suggests that Bg fibers do not contribute to the initial formation of fissures, but play an important role in the function of the anchoring centers by directing migration of gcs at the base of the fissure in a semicircle. Furthermore, this spreading out of the gcs could account for the thinner IGL at the base of the fissures.

### An alteration in the time when two anchoring centers form underlies the altered vermis foliation pattern in *Engrailed2 *mutants

**Figure 8:**
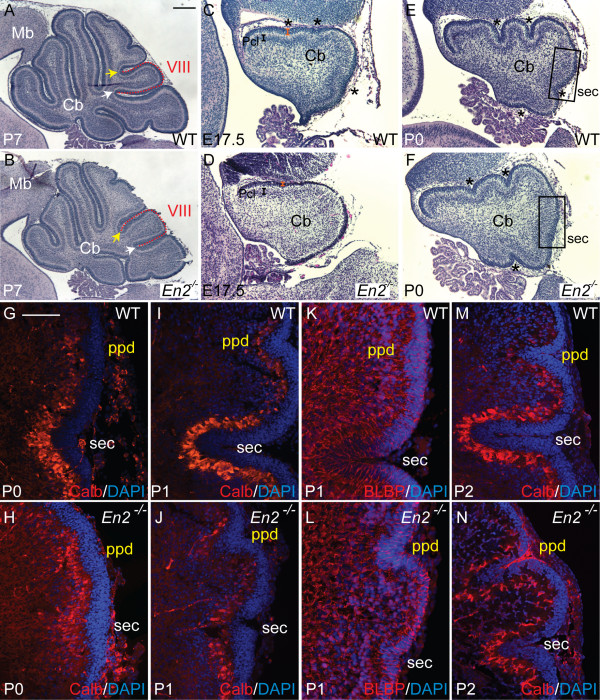
In *En2 *mutants, the timing of the formation of two anchoring centers is altered. **(a) **Midsagittal section of P7 wild-type (WT) Cb shows a normal foliation pattern. **(b) **The Cb of *En2 *mutants at P7 displays the final vermis foliation defect. Lobule VIII (red outline) is associated with the lobule IX rather than with lobules VI/VII, the secondary fissure (white arrow) is shallower than in the WT and the prepyramidal fissure (yellow arrow) is longer than in the WT. **(c, d) **Midsagittal section of an E17.5 WT and *En2 *mutant Cb shows that, only in the WT, the Pcl invaginates in the areas where three of the four future principal fissures will form (black asterisks). The orange bracket indicates the thicker EGL in the wild-type Cb. The black bracket demarcates the Pcl. **(e) **Sagittal section of a WT Cb at P0 shows the four principal fissures (black asterisks). **(f) **In a sagittal section at P0 of an *En2 *mutant Cb, only three shallow principal fissures (black asterisks) are apparent; the secondary (sec) fissure is absent. **(g-l) **Anti-Calbindin immunostaining (red) and DAPI staining (blue) of WT (g, i, m) and *En2 *(h, j, n) mutant Cb at P0 (g, h), P1 (i, j), and P2 (m, n) show a delay in formation of the secondary (sec) fissure and premature formation of the prepyramidal (ppd) fissure. Medial sagittal sections of P1 WT (k) and *En2 *mutant (l) embryos with anti-BLBP immunostaining show that Bg fibers point to an anchoring center in both secondary and prepyramidal fissure. Scale bars: (a, b,) 25 μm; (c-f) 100 μm; (g-n) 50 μm.

Our results indicate that a precise coordination of distinct behaviors of Pcs, gcps, and Bg fibers produces anchoring centers that function as the base of each fissure. By extrapolation, the position and timing of formation of each anchoring center should determine the shape and organization of the folia. To test this hypothesis, we examined a mouse mutant with an altered foliation pattern to determine whether a change in the timing and/or positioning of the key cellular events responsible for initiation of fissures occurs in accordance with the foliation defect. We chose to analyze mice lacking the gene encoding the homeobox transcription factor Engrailed2 (En2), since in the vermis of *En2 *mutants the secondary principal fissure (which normally separates the central lobe from the posterior lobe) is shifted posterior such that lobule VIII of the central lobe is associated with lobule IX of the posterior lobe rather than with lobules VI/VII of the central lobe [48]. In addition, the relative lengths of the secondary and prepyramidal fissures are reversed (Figure 8a, b) [48-52]. The specific and reproducible alteration in only one folium of *En2*^-/- ^mutants affords the opportunity to study the formation of the two anchoring centers surrounding lobule VIII amidst others that produce normal folia. We therefore examined whether the vermis foliation phenotype in *En2 *mutants is associated with a change in the timing and/or positioning of the secondary and prepyramidal anchoring centers that surround lobule VIII.

**Figure 9:**
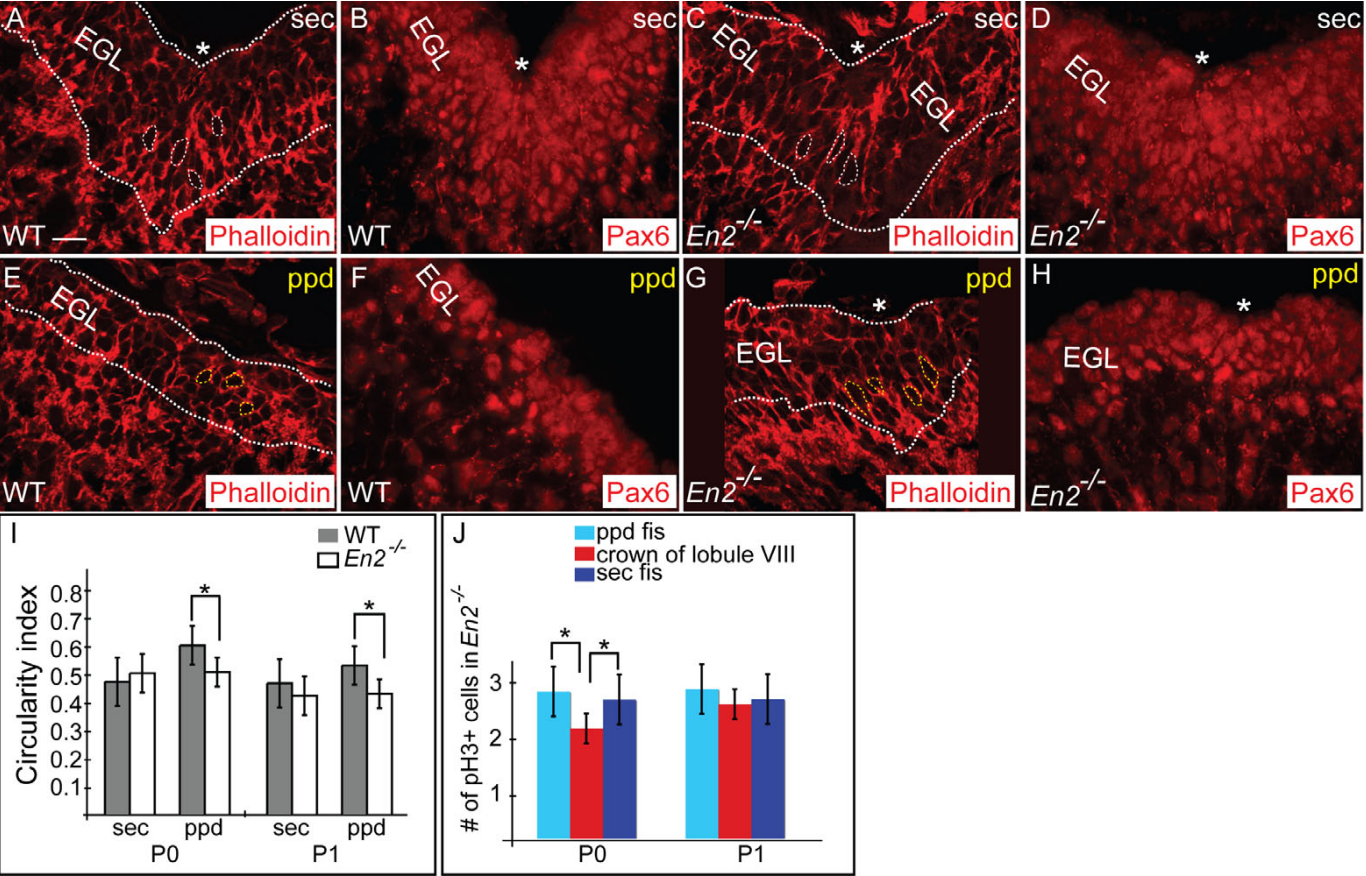
Gcps of two altered anchoring centers in mutants exhibit the same coordinated changes as in WT. **(a, c, e, g) **Phalloidin staining (red) shows gcp morphology in sagittal sections of P0 WT (a, e) and *En2 *mutant mice (c, g). At P0, in the WT secondary fissure (a), gcps are more elongated (ci = 0.55) than gcps in the area of the prepyramidal fissure (e) (ci = 0.71). In contrast, in *En2 *mutants, the gcps in the secondary (c) and prepyramidal (g) fissures are similarly elongated. **(b, d, f, h) **Dotted white line outlines the EGL, based on the anti-Pax6 immunostaining of adjacent sections to depict the thickness of EGL. Asterisks indicate the base of the fissure. **(i) **Ci of gcps (gray is WT, white is mutant). Bar height indicates the mean value of each data set, and error bars indicate standard error. An asterisk indicates statistically significant differences between the base of the fissure and crown of the folia for each data set (*p *< 0.0009 for P0; *p *< 0.0002 for P1). **(j) **Anti-pH3 immunostaining at P0 reveals that there are more gcps in mitosis in the areas where the prepyramidal and secondary fissures will form than at the crown of folium VIII. Bar graphs depict quantification of the number of pH3 positive gcps in the prepyramidal fissure (light blue bars) and in the secondary fissure (dark blue bars) compared to the crown of the intervening folium (red bars). Bar height indicates the mean value of each data set, and error bars indicate standard error. An asterisk indicates statistically significant differences between the two regions (*p *< 0.004 for P0 for the prepyramidal fissure; *p *< 0.05 for P0 for the secondary fissure). Scale bar: 15 μm.

At E17.5, the overall size of the *En2 *mutant Cb was reduced, the surface of the Cb smooth rather than having three principal fissures, and the EGL thinner than normal (Figure 8c, d), consistent with our previously documented general postnatal delay in foliation and an overall reduction in the size of the mutant adult Cb [50]. Furthermore, we found that at E17.5 the Pc layer of *En2 *mutants lacked invaginations (data not shown) and there were no signs of accumulation of gcps. An observed weak Calbindin immunostaining in *En2 *mutants might further reflect a general developmental delay in Pc maturation (data not shown). Interestingly, by P0 all the principal fissures except the secondary fissure were clearly visible in *En2 *mutants (Figure 8e, f). The Pc layer had, however, begun to fold and gcps accumulated inwards where the secondary fissure was expected to form. Moreover, despite the overall delay in formation of fissures in *En2 *mutants, the Pc layer had already begun to invaginate and gcps to accumulate at P0 in the area where the prepyramidal fissure should form, a few hours earlier than in wild-type mice (Figure 8g, h). At P1 the prepyramidal fissure of *En2 *mutants was deeper than the newly forming secondary fissure, whereas in wild-type mice the secondary fissure is much deeper than the prepyramidal fissure (Figure 8i, j). Furthermore, the Pcs found in the secondary fissure in *En2 *mutants at P1 were smaller in size and less mature than normal, whereas Pcs found in the prepyramidal fissure were more mature than Pcs found in the wild-type prepyramidal fissure (Figure 8i, j). By P2, the prepyramidal fissure had continued to lengthen in *En2 *mutants more than in wild-type mice, and the secondary fissure remained shorter than normal (Figure 8m, n). Additionally, altered timing of the organization of Bg fibers correlated with the altered fissure formation in *En2 *mutants (Figure 8k, l, and data not shown). It was not until P1 in *En2 *mutants when the prepyramidal and secondary fissures had formed that the Bg fibers fanned out from the two anchoring centers (Figure 8k, l). Consistent with the apparent altered timing of formation of the prepyramidal and secondary anchoring centers in *En2 *mutants, at P0 and P1 the gcps in the emerging secondary fissure of *En2 *mutants were elongated more than gcps between the fissures, but slightly less than normal (wild type: ci = 0.55 at P0 ci = 0.52 at P1 (Figure 9a, i, and data not shown); *En2 *mutant: ci = 0.59 at P0, ci = 0.59 at P1 (Figure 9c, i, and data not shown) and the gcps in the anchoring center of the mutant prepyramidal fissure were more elongated (ci = 0.59 at P0, ci = 0.5 at P1; Figure 9g, i) than normal (ci = 0.71 at P0, ci = 0.62 at P1, *p *< 0.0009; Figure 9e, i, and data not shown). Moreover, at P0 in *En2 *mutants quantification of pH3 positive gcps (Figure 9j) revealed that pH3 positive gcps were more frequently found in the emerging secondary and prepyramidal fissure then in the crown of the lobule between these two fissures. Our results demonstrate that in the prepyramidal and secondary anchoring centers of *En2 *mutants the normal procession of morphogenetic behaviors of Pcs, gcps and Bg fibers occurs in unison with the premature formation of the prepyramidal fissure and delayed formation of the secondary fissure compared to the same wild-type fissures. This altered timing of anchoring center formation then leads to the apparent posterior shift of lobule VIII.

## Discussion

**Figure 10:**
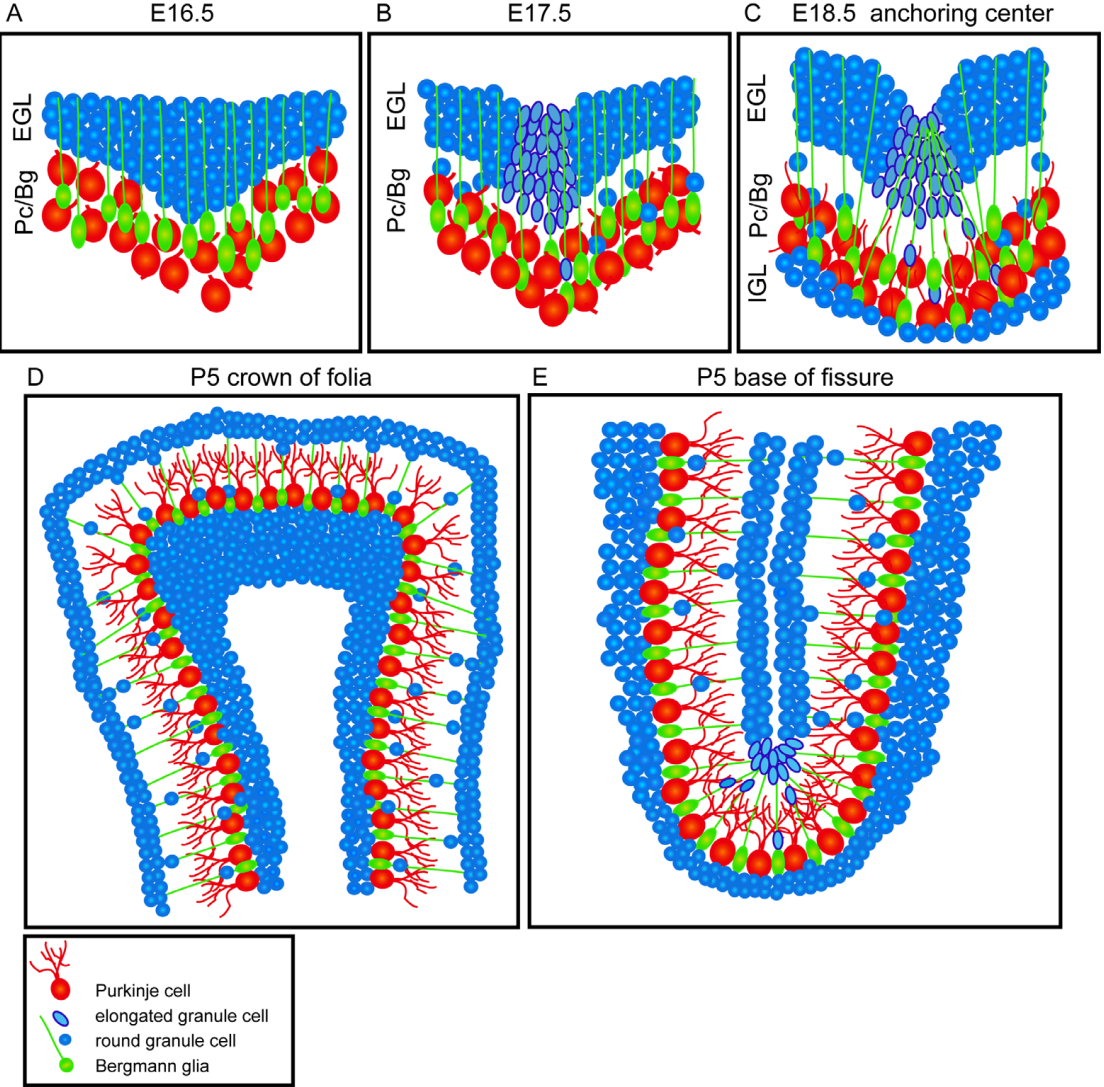
Mouse cerebellar foliation is initiated by the formation of multi-cellular anchoring centers. **(a) **At E16.5, the first stage of anchoring center formation is an inward accumulation of gcps (blue) resulting from increased proliferation. The Pc layer (red) simultaneously folds inward, precisely where the gcps accumulate. **(b) **Next, at E17.5, gcps elongate and align along the fissure, as the outer cerebellar surface begins to fold inwards **(c) **By E18.5, the Bg fibers near the anchoring centers converge on the base of the fissure. **(d, e) **Cerebellar foliation continues by a self-sustaining process of folial outgrowth. (d) Bg fibers in the anchoring centers fan out from a small source to cover a large area of the IGL, thus forming a thin IGL at the base of the fissure. (e) Conversely, granule cells converge on a smaller central area and, therefore, accumulate at the crown of the folia to promote folial lengthening.

Collectively, our analyses provide a new model for the initiation of fissures and growth of cerebellar folia (Figure 10). The first indication of emerging fissures in the vermis is an inward accumulation of gcps due to increased proliferation. Most likely as a consequence, the Pc layer folds inwards at the nodes of gcp accumulation. Subsequently, gcps in the accumulations change their cell shape from round to more elongated and align along the axis of the fissure. As the outer cerebellar surface begins to fold inwards, the Bg fibers converge at the base of the fissure. Finally, gcps preferentially differentiate within the emerging fissures. Based on staining with markers for gcps, Pcs and Bg fibers, the same coordinated morphological changes in these three cell types also occur where the fissures emerge and become the base of the fissures in the hemispheres (Additional file 3). We therefore propose to call these regions 'anchoring centers' for the foliation process. Furthermore, once the anchoring centers are established, we suggest that outward lengthening of folia is driven by a self-sustaining method involving gcp proliferation and directed migration of post mitotic gcs by Bg fibers. Finally, the size and shape of the folia is determined largely by the time and position at which each anchoring center forms.

### Establishment of anchoring centers is associated with increased granule cell precursor proliferation and cell shape changes

Studies in both the rat and mouse Cb have demonstrated a requirement for gcp proliferation in order for foliation to proceed. For example, the experimental reduction of gcps in the postnatal rat Cb using irradiation or a genetically engineered decrease in gcp proliferation as a result of mutations in components of the Shh signaling pathway in mice leads to premature depletion of the EGL and an immature (less complex) foliation pattern [13,53]. Conversely, prolonged proliferation of gcps in the rat EGL due to hypothyroidism or in transgenic mice due to excess Shh signaling results in formation of additional folia [13,54]. It was not clear from these studies, however, whether gcp proliferation drives initiation of Cb foliation.

Our studies have revealed that an increase in the mitotic index of gcps and an associated inward thickening of the EGL are the first signs of fissure formation at E16.5, before Shh is expressed by Pcs. Furthermore, previous studies have shown that once Shh is expressed at E17.5, while it is a critical mitogen for gcps there is no evidence that Shh signaling is higher at the anchoring centers [12,13,55,56]. The second unique feature of gcps in emerging fissures is an elongation of their cell bodies parallel to the fissure at E17.5. It is worth noting that it is unusual for neural progenitors to be elongated when they are dividing. Since the cells do not express Tau (Tau-myrGFP) and the axis of elongation is 90° to that of the migrating gcs, it is unlikely that the elongated cells have begun migrating. Instead, this change in gcp cell shape could drive fissure formation since in other systems it has been demonstrated that changes in cell shape precede groove formation. For example, an elongation in cell shape drives the formation of the morphogenetic furrow in the *Drosophila *eye imaginal disc and neuroepithelial cells at the base of the avian neural plate become wedge-like to allow bending of the neural tube [57,58]. Moreover, an intimate link between the plane of cell division and changes in cell shape has been shown to be necessary for neural tube formation in zebrafish [59,60]. This raises the question of whether oriented cell division in addition to cell shape changes play a role in initiation of fissure formation at anchoring centers.

The confinement of morphological changes in gcps to the base of fissures suggests that intrinsic genetic patterning programs create differences between the gcps at the base of fissures (anchoring centers) from the remaining gcps. One previous study in chick suggested that gcps found at the crown of the folia are genetically different from gcps at the base of the fissures, since the latter specifically express the homeobox-containing transcription factor Tlx-3 [61]. However, the mouse homolog of *Tlx-3 *(*Rnx*) is not spatially restricted to the base of fissures but is instead confined to gcps in the central and posterior lobe at P0 [62] (AS and ALJ, unpublished observation). Thus, if there are universal molecular factors that are responsible for orchestrating the cellular events that result in local gcp shape changes and increased proliferation, they have yet to be identified. Gene expression studies comparing gcps within anchoring centers versus gcps in the rest of the Cb would provide a possible means to identify such critical factors.

### Folding of the Pc layer occurs in unison with the inward accumulation of gcps during establishment of anchoring centers

In addition to the inward accumulation of gcps at E16.5 where the fissures will form, we observed a complementary in-folding of the Pc layer at the same time and position. Unlike the gcps, however, the Pcs do not appear to take on a distinct cell shape or orientation at the base of fissures until after birth. Thus, it is likely that the inward accumulation of gcps is the driving force for the folding of the Pc layer, and possibly for fissure formation itself.

The fact that the EGL forms an invagination rather than evagination of the Cb surface raises the question of whether gcps actively push inwards, or whether forces within the Cb dictate the contour of the cell accumulation. One possible explanation involving mechanical forces is that the Cb cortex has less inherent force resistance than the outer surface, which is made up of a basal lamina and an ependymal cell layer and, thus, the gcps take the path of least resistance. An attractive hypothesis whereby the changes in gcps and Pcs could be coupled is that a subset of Pcs secrete an as yet to be identified factor locally that increases gcp proliferation and induces cell elongation, and that these changes in gcps then produce the inward folding of the Pc layer and subsequently the outer surface of the Cb. Our study is the first to demonstrate that coordinated changes in the Pc layer and gcp behaviors are the first cellular signs of sites where the outer surface of the Cb will form fissures.

### Bergmann glia fiber orientation and distinctive granule cell migration produce functional anchoring centers that drive folial outgrowth

Our studies indicate that after accumulation and elongation of gcps, the final process of establishing a functioning anchoring center is the specific reorganization of the Bg fibers into a fan shape at the base of the fissures at E18.5. As the Cb surface foliates, we found that the Bg fibers surrounding the anchoring center change from a parallel organization to radiate in to the pial surface of the anchoring center, resembling spokes radiating out from the hub of a wheel. It is possible that such a rearrangement of Bg fibers, similar to the drawing up of a purse string, involves an active process intrinsic to the Bg. Alternatively, rearrangement of Bg fibers may be a mechanical consequence of fissure formation in the cerebellar surface that is driven by the elongation of gcps (our studies) or the proposed restraint of Pc axons [9].

Since Bg fibers serve as trajectories along which gcs migrate from the EGL to the IGL, the fan-like organization of Bg fibers at the base of a fissure likely directs the gcs surrounding the anchoring center to disperse around the whole base of the fissure, which would produce the thinner IGL observed at the base of fissures [39,41]. We propose that once an anchoring center is set up, the rest of folial outgrowth proceeds in a self-sustaining manner. Since the gcps at the base of the fissure must fill a much larger area of the IGL than gcps elsewhere, the base of the fissure would be expected to undergo little outward expansion. Furthermore, the opposite should occur at the crown of the folia where gcps merge into a smaller area of the IGL, thus resulting in outward growth and a thicker IGL at the crown of folia. Gcps along the sides of the fissures migrate directly across the molecular layer and, thus, should expand the length of the folia. The likely importance of Bg as a critical component of a functional anchoring center has recently been indicated by a mutant in which the Bg fail to mature due to ectopic expression of *Sox4 *[63]. Although there are local accumulations of gcps in these mutants that are accompanied by invaginations of the Pc layer, fissure maturation does not proceed.

### Alterations in the timing of anchoring center formation results in predictable changes in folial shape

**Figure 11:**
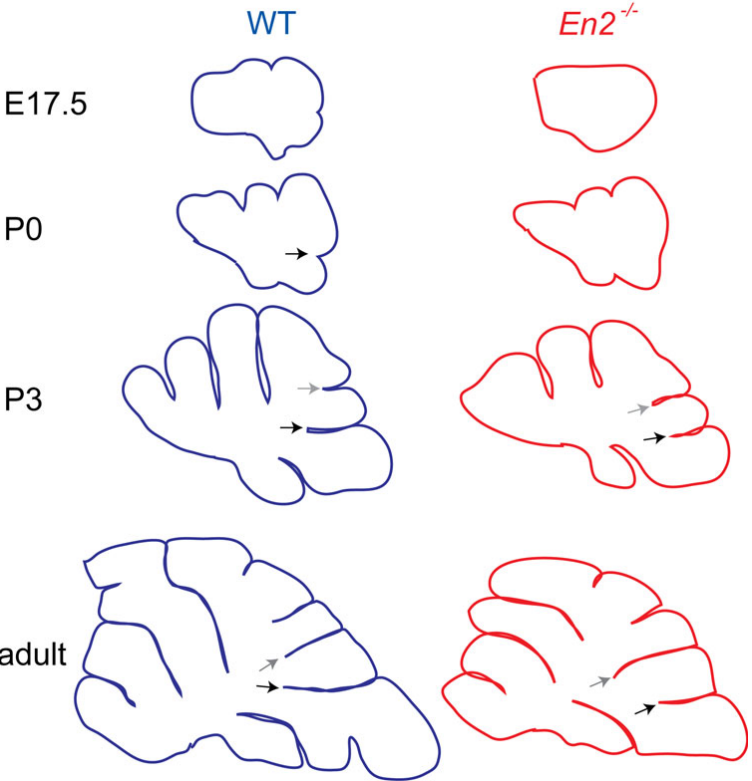
Correct timing of anchoring center initiation is required for correct folial shape. Superimposition of midline vermis tracings at E17.5, P0, P3 and adult show that the delay in anchoring center formation of the secondary fissure and premature anchoring center formation of the prepyramidal fissure in *En2 *mutants result in the a misshapen lobule VIII. Grey arrows demarcate prepyramidal fissure; black arrows demarcate secondary fissure.

Our analysis of fissure formation of *En2 *mutants not only provides evidence for our proposed sequence of cellular events that dictate anchoring center formation but, importantly, also reveal that the timing of anchoring center formation plays a critical role in determining the shape of folia (Figure 11). We found that all of the changes in gcps, the Pc layer and Bg fibers associated with anchoring center formation are initiated prematurely in the prepyramidal (non-principal) fissure, whereas those in the secondary (principal) fissure are considerably delayed in *En2 *mutants (Figure 11). Our findings in *En2 *mutants suggest the existence of a morphogenetic clock that normally controls the timing of the genetic and cellular events that direct formation of anchoring centers. This morphogenetic clock must ensure that the cellular behaviors associated with anchoring center formation occur at the right time. It appears that the loss of *En2 *disturbs this morphogenetic clock, leading to aberrations in the timing of formation of the prepyramidal and secondary fissures. This leads to an alteration in the size, shape and position of the intervening lobule VIII. It is not clear whether *En2 *specifically affects Pcs in the anchoring centers responsible for generating lobule VIII, which in turn fail to provide local factors for proliferation and elongation of gcps, and/or whether *En2 *directly regulates gcp or Bg behavior. Cell type specific ablation of *En2 *could be used to further dissect its role in each cell type.

## Conclusion

Our findings unite and broaden the proposed models for Cb foliation to encompass synchronization of multiple distinct cellular behaviors in the initial formation of fissures and subsequent growth of folia. As such, we propose that the base of fissures are genetically and morphologically distinct regions of the Cb that not only anchor the base of the fissures but also drive the outward growth of the lobules. Furthermore, both the position and time at which each of the anchoring centers form must be precisely regulated in order to produce a normal pattern of folia. We suggest that the extent of the array of folia in each species provides part of the basis for organizing the range of functional circuits in an organism. Moreover, it is conceivable that during evolution the number of folia can increase through balancing an increase in gcp proliferation with addition of extra anchoring centers in particular positions. The distinct shape of each folium would then evolve through changing the timing of when the anchoring centers form. Finally, the fact that in *En2 *mutants the timing and positioning of fissure formation is uncoupled indicates that the evolutionarily conserved genetic information that dictates where the anchoring centers should form functions independently of the processes governing when the Pcs, gcps, and Bg respond to this information and execute anchoring center formation.

## Materials and methods

### Mouse lines

All animal studies were carried out in outbred SW mice, under an approved IACUC animal protocol according to the institutional guidelines at New York University School of Medicine and Memorial Sloan-Kettering Cancer Center. In almost all SW mice analyzed, the most anterior lobules I and II were fused, and lobules IV and V were partially separated by a fissure. Sublobules VIa and b, as well as IXa, b, and c, were always present. The day that a plug was detected was designated as E0.5. The day of birth was designated as P0. Adults were designated as P28 or older. For cell shape analysis transgenic *CAG::GPI-eGFP *mice were used [29]. *Tau-loxP-STOP-loxP-myrGFP-IRES-nLacZ *(*Tau*) reporter mice were genotyped as described [35]. *Math1-CreER *mice were genotyped as described [34]. For genotyping the *En2 *mutant allele, the following primers were used p32 (5'-TCGGGGGAAGAAGTGTCCAATGTCC-3'), neoTGAp2 (5'-ATCGCCTTCTTGACGAGTTCTTCTGAG-3') and En2p31 (5'-GGGCCTGTACAACCATTCCACCACG-3') [49]. The p31 and p32 primers amplify the wild-type band.

### Fate mapping

Double hemizygous males (*Math1-CreER; Tau-loxP-STOP-loxP-myrGFP-IRES-nLacZ*) were bred with SW females (five to six weeks old; Taconic, Hudson, NY, USA) to generate double hemizygous embryos. Tamoxifen (T-5648, Sigma, St. Louis, MO, USA) was dissolved in corn oil (Sigma C-8267) at a final concentration of 20 mg/ml. The females were given tamoxifen via gavage with animal feeding needles (Fine Science Tools, Corston, UK) at noon on E15.5 (4 mg per 40 g of body weight). Double hemizygous males (*R26-CreER; R26-loxP-STOP-loxP-eYFP*) were bred with SW females (five to six weeks old; Taconic) to generate double hemizygous embryos. The females were given tamoxifen at noon on E12.5 (1–2 mg per 40 g of body weight) and analyzed at E16.5/17.5. Dissected brains were immersion fixed for 20 minutes in 4% paraformaldehyde (PFA) at 4°C, cryoprotected in 15% and 30% sucrose, embedded in OCT (Tissue-Tek, Sakura Finetechnical, Japan) and sectioned at 10 μm.

### Histology and immunofluorescent immunohistochemistry

For histology and immunofluorescent immunohistochemistry of mice older than P8, brains were dissected after intracardiac perfusion with 4% PFA and then immersion fixed in 4% PFA overnight at 4°C. Tissue was then processed for paraffin embedding and sectioned at 7 μm. For consistency, sections analyzed from the vermis were limited to the most medial 100–200 μm.

Immunohistochemistry using indirect immunofluorescence was performed using standard staining procedures with the following antibodies: rabbit anti-BLBP (1:2,000; Milipore, Billerica, MA, USA), mouse anti-BrdU (1:500; Becton Dickinson, Franklin Lakes, NJ, USA), mouse anti-Calbindin (1:4000; Swant, Bellizona, Switzerland), mouse anti-NeuN (1:1,000; Chemicon, Temecula, CA, USA), rat anti-GFP (1:5,000; Nacalai Technique, Kyoto, Japan), rabbit anti-GFP (1:5,000; Invitrogen Corporation, Carlsbad, CA, USA), rabbit anti-PH3 (1:500; Cell Signaling Technology, Beverly, MA, USA), anti-βgal (1:500; Biogenesis, Raleigh, NC, USA), rabbit anti-Pax6 (1:300; Chemicon), mouse anti-GFAP (1:500; Chemicon), TRITC-Phalloidin (1:2,000; Sigma), goat anti-Sema6a (1:200; R&D Systems, Minneapolis, MN, USA). Sections were mounted in Vecta Shield with DAPI (Vector Laboratories, Burlingame, CA, USA) and examined with a fluorescent microscope (DM6000, Leica, Nussloch, Germany; Axio Observer, Zeiss, Germany). Fluorescent images for Figure 2g–g2 were captured in 1.5 μm optical sections using Zeiss Observer with Apotome setting and processed using Adobe Photoshop. Orthogonal analysis was performed to confirm co-expression of two markers.

The Cb tracings of midline sagittal sections of the vermis were done at E16.5, E17.5, E18.5, P3, P5, P7, P10 and P21 by photographing hematoxylin and eosin sections using a dissection microscope (MZ16FA, Leica) at 1× magnification, followed by careful outline of the outer most surface using Adobe Illustrator (CS2) and then overlaying the outlines on top of each other.

### Bromodeoxyuridine staining and quantification of cell proliferation

To assay proliferation, pregnant females were injected intraperitoneally with 100 μg BrdU/g body weight 20 minutes before they were sacrificed. To quantify the percentage of BrdU-positive cells at E17.5 and E18.5, the percentage of BrdU-positive cells was calculated by counting the total number of cells in the EGL (DAPI positive cells) of 40–50 sections (7 μm thick) per embryo from the most medial 100–200 μm of 3 embryos at each stage. To determine the distribution of pH3 positive cells in the EGL, the number of pH3 positive cells at the base of each fissure versus the side and crown of the folia was counted at E16.5, E17.5 and E18.5. The base of the fissure was considered to be the most invaginated part of the fissure. The percentage of pH3-positive cells was then calculated from the number of pH3-positive cells per total number of granule cell precursors in each region. The total number of gcps in each region was calculated by counting the number of nuclei based on DAPI staining. The crown of folia had 100–200 nuclei at E16.5, 120–276 nuclei at E17.5, and 100–280 nuclei at E18.5. The base of fissure had 80–90 nuclei at E16.5, 100–144 nuclei at E17.5, and 100–150 nuclei at E18.5. The quantification of pH3 positive cells was done for 45 to 60 of the most medial consecutive sections (7 μm thick) of three brains for each embryonic stage (E16.5-E18.5). For *En2 *mutants at P0 and P1 the quantification of pH3 positive cells was done for the 25–30 most medial consecutive sections (7 μm thick) of three brains for each stage. The quantification of BrdU and βgal double positive cells was done for seven to eight of the most medial sagittal consecutive frozen sections (12 μm thick) of three brains at E18.5. Measurements of cells from multiple embryos were pooled into datasets. We defined the area of the base of the fissure in which positive cells were counted to be the most invaginated part of the fissure and an underlying fan shaped area that is 10 μm deep under the EGL (see Fig. 6f). We defined the area of the crown of the fissure in which positive cells were counted to be the same length of EGL as in the base of the fissure but at the top of the adjacent lobule and a 10 μm deep rectangle below this (yellow inset in Figure 6f).

### Morphometrics

We used ImageJ software from National Institutes of Health (NIH) to trace cell outlines and measure cell perimeters [64]. Cells were chosen for quantification only if their outline and morphology was clearly visible. Circularity was calculated by ImageJ as a normalized ratio of area (A) to perimeter (P), with a ratio of 1 representing a circle (circularity = 4πA/P^2^). By performing these calculations, circularity index distinguishes cells with round morphologies from those with more elongated morphologies.

To quantify the shape of gcps found in the bottom of the fissure and the crown of the lobe, high magnification images were taken of mid-sagittal Cb sections of *CAG::GPI-eGFP *transgenic mice at E16.5, E17.5 and P0. For quantification, we used 40–50 sections (7–10 μm thick) total from the most medial 300 μm of 3 embryos at each stage. Based on anti-Pax6 and anti-GFP double immunostaining, we determined that the five cell thick layer at the crown of the folia, and seven cell thick layer at the base of the fissures is the EGL and confined our measurements to this layer. Measurements of cells from multiple embryos were pooled into datasets. Sample sizes were as follow: smooth Cb at E16.5 from 3 embryos, 60 cells distributed along the AP axis of the Cb and 45 cells found in the area where EGL accumulated; the base of fissures at E17.5, 63 cells from 3 embryos; the crown of the lobes at E17.5, 70 cells from 3 embryos; the base of fissures at P0, 116 cells from 3 embryos; the crown of the lobes at P0, 125 cells from 3 embryos. For *En2 *mutant animals we calculated the circularity index for the outermost five cell thick layer of the cerebellar cortex. The sample sizes were as follows: both secondary and prepyramidal fissure at P0, 20–30 cells for 3 embryos; at P1, 30–35 cells from 3 embryos. Circularity index values were compared using unpaired *t*-tests.

### Electron microscopy

E17.5 embryos were perfused with 4% PFA followed by an immersion fixation in 3% paraformaldehyde, 1% glutaraldehyde, 4% sucrose, 0.1% CaCl_2 _and 2.5% DMSO in 0.1 M sodium cacodylate buffer (pH 7.4). The Cb was removed and again immersion-fixed at 4°C in 4% PFA for 2 hours and post fixed with 1% osmium tetroxide at room temperature for 1.5 hours, then processed in a standard manner and embedded in Embed 812 (EMS, Hatfield, PA, USA). Semi-thin sections were cut at 1 μm and stained with 1% toluidine blue to evaluate preservation quality. Ultrathin sections (60 nm) were cut using a Leica ultracut UCT, put on formover coated copper grids and stained with uranyl acetate and lead citrate by standard methods. Stained grids were examined under a Philips CM-12 electron microscope, and photographed with a Gatan 1 k × 1 k digital camera.

## Competing interests

The author(s) declare that they have no competing interests.

## Authors' contributions

AS and ALJ designed the experiments and AS performed all of the experiments. The manuscript was written by AS and ALJ. AS and ALJ read and approved the final manuscript.

## Supplementary Material

Additional file 1 Initially a diffuse layer, the IGL remains thinnest at the base of each fissure. The data provided represent marking of the IGL in the early postnatal cerebellum. **(a) **Anti-NeuN staining reveals a loosely organized IGL at P1, both at the base and crown of the lobes. **(a1) **A higher magnification of area indicated in (a). **(b) **By P3, the IGL appears as a distinct layer that is thinnest at the base of the fissures (b1) and thickest at the crown of the lobes (b2). **(c) **Anti-p27 staining reveals the compact organization of the IGL at P7. The IGL is much thinner at the base of the fissures (c1) than on the sides of the lobes and thickest at the crown of the lobes (c2). p27 labels post-mitotic granule cells in the iEGL and IGL. Scale bars: (a, a1) 100 μm; (b, c) 300 μm; (b1, b2, c1, c2) 75 μm. Click here for file

Additional file 2 In the outermost layer of Cb cortex, BrdU positive cells are granule cell precursors. The data provided represent labeling for BrdU and Pax6 and show that a 20 minute pulse of BrdU marks granule cell precursors in the EGL. Medial sagittal sections of **(a) **E16.5, **(b) **E17.5 and **(c-e) **E18.5 embryos treated for approximately 20 minutes with BrdU. Double immunostaining with anti-BrdU (green) and anti-Pax6 (red) show uniform BrdU incorporation throughout the EGL. Scale bars: (a, c) 50 μm; (b, d, e) 15 μm. Click here for file

Additional file 3 Same morphological changes in gcps, Pcs and Bg fibers occur in emerging fissures in hemispheres as in the vermis. The data provided represent marking of different cell types to highlight the morphological changes in gcps, Pcs, and Bg fibers in the hemispheres. Sagittal sections of **(a-d) **E17.5 and **(e-h) **E18.5 hemisphere stained for gcp, Pc and Bg fiber markers. (a, e) DAPI staining reveals a smooth surface in the lateral Cb. Anti-Calbindin (b, f) and anti-Pax6 (c, g) immunostaining shows that the Pc layer invaginates, and gcps accumulate in the areas where fissures will form. (d) At E17.5, anti-GFP immunostaining of *CAG::GPI::GFP *lateral sections shows both round and more elongated shaped gcps at newly emerging anchoring centers. (h) Anti-BLBP (red) and anti-BrdU (green) immunostaining of lateral section of E18.5 reveals that Bg fibers remain parallel to each other since the outer surface is still smooth. Scale bars: (a-c, e-g) 100 μm; (d, h) 15 μm. Click here for file
