# Supplementary figures and images for: Cerebellum morphogenesis: the foliation pattern is orchestrated by multi-cellular anchoring centers
Source: Neural Dev. 2007 Dec 3;2:26. doi: 10.1186/1749-8104-2-26 (PMC2246128; doi:10.1186/1749-8104-2-26)

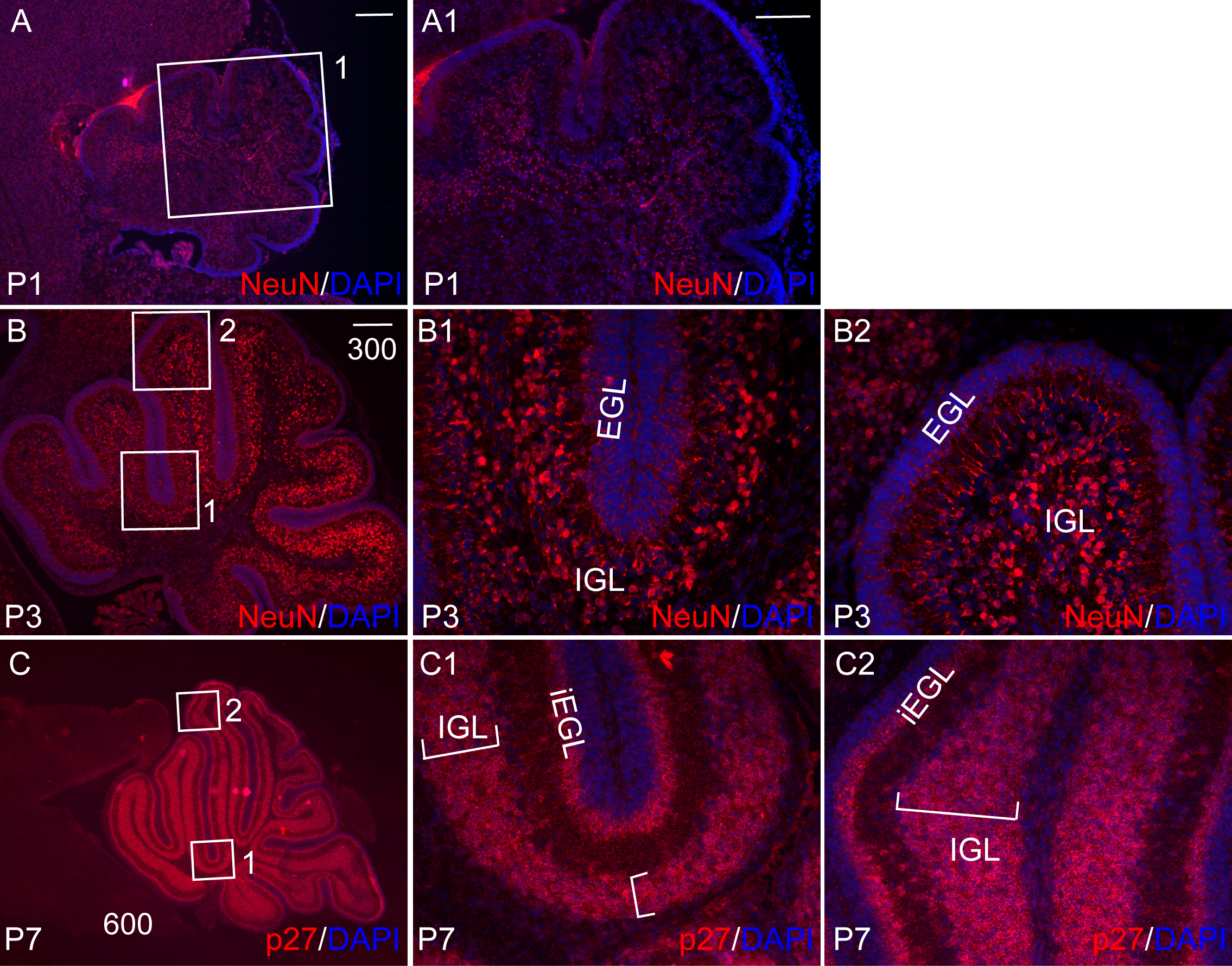

Supplement: Additional file 1 — Initially a diffuse layer, the IGL remains thinnest at the base of each fissure. The data provided represent marking of the IGL in the early postnatal cerebellum. (a) Anti-NeuN staining reveals a loosely organized IGL at P1, both at the base and crown of the lobes. (a1) A higher magnification of area indicated in (a). (b) By P3, the IGL appears as a distinct layer that is thinnest at the base of the fissures (b1) and thickest at the crown of the lobes (b2). (c) Anti-p27 staining reveals the compact organization of the IGL at P7. The IGL is much thinner at the base of the fissures (c1) than on the sides of the lobes and thickest at the crown of the lobes (c2). p27 labels post-mitotic granule cells in the iEGL and IGL. Scale bars: (a, a1) 100 μm; (b, c) 300 μm; (b1, b2, c1, c2) 75 μm. [file 1749-8104-2-26-S1.tiff]

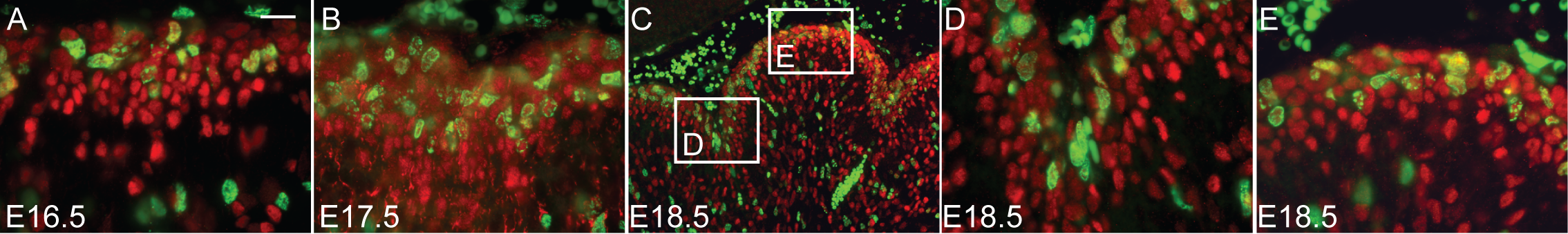

Supplement: Additional file 2 — In the outermost layer of Cb cortex, BrdU positive cells are granule cell precursors. The data provided represent labeling for BrdU and Pax6 and show that a 20 minute pulse of BrdU marks granule cell precursors in the EGL. Medial sagittal sections of (a) E16.5, (b) E17.5 and (c-e) E18.5 embryos treated for approximately 20 minutes with BrdU. Double immunostaining with anti-BrdU (green) and anti-Pax6 (red) show uniform BrdU incorporation throughout the EGL. Scale bars: (a, c) 50 μm; (b, d, e) 15 μm. [file 1749-8104-2-26-S2.tiff]

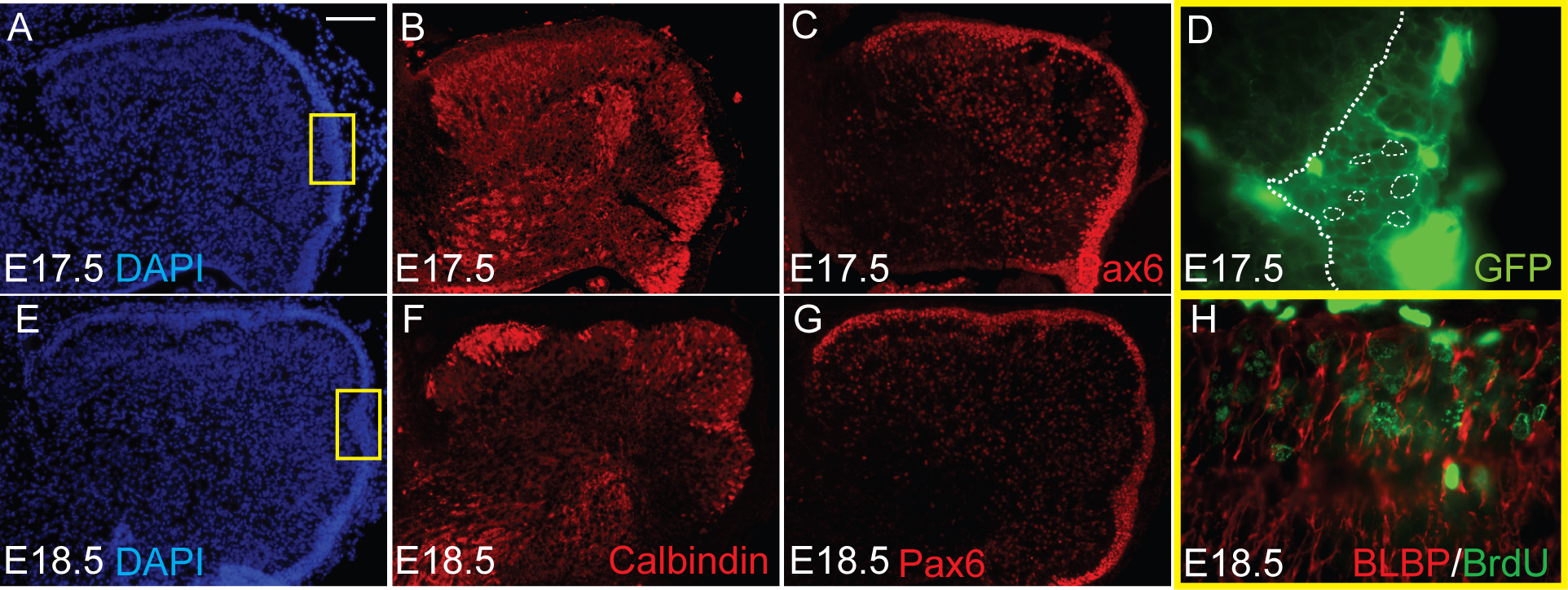

Supplement: Additional file 3 — Same morphological changes in gcps, Pcs and Bg fibers occur in emerging fissures in hemispheres as in the vermis. The data provided represent marking of different cell types to highlight the morphological changes in gcps, Pcs, and Bg fibers in the hemispheres. Sagittal sections of (a-d) E17.5 and (e-h) E18.5 hemisphere stained for gcp, Pc and Bg fiber markers. (a, e) DAPI staining reveals a smooth surface in the lateral Cb. Anti-Calbindin (b, f) and anti-Pax6 (c, g) immunostaining shows that the Pc layer invaginates, and gcps accumulate in the areas where fissures will form. (d) At E17.5, anti-GFP immunostaining of CAG::GPI::GFP lateral sections shows both round and more elongated shaped gcps at newly emerging anchoring centers. (h) Anti-BLBP (red) and anti-BrdU (green) immunostaining of lateral section of E18.5 reveals that Bg fibers remain parallel to each other since the outer surface is still smooth. Scale bars: (a-c, e-g) 100 μm; (d, h) 15 μm. [file 1749-8104-2-26-S3.tiff]
